# High frequency of HTLV-1 infection in Bantus and Pygmies from rural Cameroon bitten by non-human primates during hunting

**DOI:** 10.1186/1742-4690-11-S1-P57

**Published:** 2014-01-07

**Authors:** Claudia Filippone, Edouard Betsem, Sylviane Bassot, Patricia Tortevoye, Alain Froment, Arnaud M Fontanet, Antoine Gessain

**Affiliations:** 1 Institut Pasteur, Unité d’Epidémiologie et Physiopathologie des Virus Oncogènes, Département de Virologie, Paris, France; 2 CNRS, UMR3569, Paris, France; 3 Faculté de Médecine et des Sciences Biomédicales, Université de Yaoundé I, Yaoundé, Cameroun; 4 Institut de Recherche pour le Développement, Musée de l'Homme, Paris, France; 5 Institut Pasteur, Unité de Recherche et d'Expertise Epidémiologie des Maladies Emergentes, Département d’Infection et Epidémiologie, Paris, France

## 

HTLV-1 infection is endemic in Central Africa, as well as the closely related STLV-1 found in several non-human primates (NHPs). Like other retroviruses, acquisition through interspecies transmission is strongly suggested but needs to be investigated. We analyzed 269 selected individuals (254 men, 15 women, average age 43.5 years) for whom a direct contact (mainly a severe bite) with a NHP occurred. This happened mostly during hunting activities and involved bleeding and body fluids exchange with at least saliva/blood contact. The same number of persons who live in the same villages/settlements but did not report any bite by NHPs was matched according to sex, age and ethnicity. Both groups include either Pygmies or Bantus living in the rain forest of South Cameroon. Plasma were tested for HTLV serology by WB, and proviral DNA was searched in buffy-coat DNA by 3 HTLV generic and one HTLV-1 specific PCR. HTLV-1 prevalence was of 8.5% (23/269) among bitten individuals *versus* 1.5% (4/269) observed in the controls (p<0.001). The 23 HTLV-1+ bitten individuals reported a gorilla (17), chimpanzee (3) or monkey (3) bite. Interestingly, 13/23 were coinfected by a simian foamyvirus, for which cross-species transmission from NHP to humans through bites is demonstrated. Moreover, familial studies excluded the other established routes of virus acquisition among some positive bitten individuals. Lastly, a phylogenetic analysis showed a HTLV-1 subtype F in a bitten subject closely related to the STLV-1 strain from a *Cercocebus agilis* with whom he was in contact, thus strengthening these new findings.

